# Development and use of a scale to assess gender differences in appraisal of mistreatment during childbirth among Ethiopian midwifery students

**DOI:** 10.1371/journal.pone.0227958

**Published:** 2020-01-16

**Authors:** Rena Bakker, Ephrem D. Sheferaw, Jelle Stekelenburg, Tegbar Yigzaw, Marlou L. A. de Kroon

**Affiliations:** 1 Department of Health Sciences, Global Health, University Medical Center Groningen, University of Groningen, Groningen, the Netherlands; 2 Jhpiego Ethiopia, Addis Ababa, Ethiopia; 3 Department of Obstetrics and Gynecology, Leeuwarden Medical Centre, Leeuwarden, the Netherlands; 4 Department of Health Sciences, University Medical Center Groningen, University of Groningen, Groningen, the Netherlands; Medical University Innsbruck, AUSTRIA

## Abstract

Mistreatment during childbirth occurs across the globe and endangers the well-being of pregnant women and their newborns. A gender-sensitive approach to mistreatment during childbirth seems relevant in Ethiopia, given previous research among Ethiopian midwives and patients suggesting that male midwives provide more respectful maternity care, which is possibly mediated by self-esteem and stress. This study aimed a) to develop a tool that assesses mistreatment appraisal from a provider’s perspective and b) to assess gender differences in mistreatment appraisal among Ethiopian final-year midwifery students and to analyze possible mediating roles of self-esteem and stress. First, we developed a research tool (i.e. a quantitative scale) to assess mistreatment appraisal from a provider’s perspective, on the basis of scientific literature and the review of seven experts regarding its relevance and comprehensiveness. Second, we utilized this scale, the so-called Mistreatment Appraisal Scale, among 390 Ethiopian final-year midwifery students to assess their mistreatment appraisal, self-esteem (using the Rosenberg Self-Esteem Scale), stress (using the Perceived Stress Scale) and various background characteristics. The scale’s internal consistency was acceptable (α = .75), corrected item-total correlations were acceptable (.24 - .56) and inter-item correlations were mostly acceptable (.07 - .63). Univariable (*B* = 3.084, 95% CI [-.005, 6.173]) and multivariable (*B* = 1.867, 95% CI [-1.472, 5.205]) regression analyses did not show significant gender differences regarding mistreatment appraisal. Mediation analyses showed that self-esteem (*a*_*1*_*b*_*1*_ = -.030, *p* = .677) and stress (*a*_*2*_*b*_*2*_ = -.443, *p* = .186) did not mediate the effect of gender on mistreatment appraisal. The scale to assess mistreatment appraisal appears to be feasible and reliable. No significant association between gender and mistreatment appraisal was observed and self-esteem and stress were not found to be mediators. Future research is needed to evaluate the scale’s criterion validity and to assess determinants and consequences of mistreatment during childbirth from various perspectives.

## Introduction

Every day, about 800 women and 7,300 newborns die from causes related to pregnancy and childbirth [1,2]. Most of these deaths are preventable and occur in low-income countries, especially in Sub-Saharan Africa [3]. Ethiopia has one of the highest numbers of maternal and newborn deaths, with a maternal mortality ratio of 353 and a neonatal mortality rate of 2,800 per 100,000 live births [4,5]. Skilled birth attendants play an essential role in reducing maternal and newborn mortality [6]. However, only 28% of births in Ethiopia were attended by health professionals in 2016, in stark contrast to the 78% globally [7,8]. A factor that may account for the underuse of maternal healthcare services in low-income settings is care providers’ mistreatment of women during childbirth, which creates fear and hesitancy in women when approaching health facilities for delivery care [9–11].

Mistreatment during childbirth is a human rights violation that can be defined as conditions and encounters experienced as humiliating or undignified [12,13]. Bohren et al. [14] created an evidence-based typology of mistreatment that describes its emergence at an interpersonal but also at a health system level and comprises seven domains: 1. physical abuse, such as slapping, 2. sexual abuse, such as rape, 3. verbal abuse, such as shouting, 4. stigma and discrimination, such as providing poor treatment due to HIV status, 5. failure to meet professional standards, such as neglect, 6. poor rapport between women and providers, such as dismissal of women’s concerns, and 7. health system conditions and constraints, such as lack of privacy. Mistreatment is often justified as a means of punishment for patients’ misbehavior [15]. It is also believed to increase women’s cooperation during childbirth, which in turn is thought to benefit the well-being of the newborn, while long-term physiological and psychological damage to its victims is often disregarded by both care providers and society [11].

Mistreatment during childbirth has been frequently observed at Ethiopian obstetric care facilities. A study that looked at mistreatment during childbirth in Ethiopia indicated that 36% of women reported at least one form of mistreatment, with a high prevalence of neglect (19%), privacy violations (17%), physical abuse (9%) and verbal abuse (8%) [16]. Moreover, research has pointed towards settings in which Ethiopian patients were not allowed to bring birth companions and deliver in their preferred birthing position, next to being subjected to inadequate pain management and being exposed to healthcare interventions, such as episiotomies, without giving their consent [17].

Previous research on mistreatment during childbirth has mostly been qualitative, which limits the generalizability of study outcomes [14]. A research tool (e.g. a quantitative scale) is needed in order to assess to what extent Ethiopian care providers are prone to mistreat their patients during labor. Recently, Sheferaw et al. [18] created a quantitative scale to assess patients' perception of mistreatment during childbirth. However, to our knowledge there is no quantitative scale that assesses care providers’ perspective of mistreatment during childbirth. Such a scale could generate generalizable results when appraising risk factors among care providers that are related to mistreatment during childbirth. This may provide sound insights for respectful maternity care (RMC) interventions, which can be of particular interest to the Ethiopian government that aims to promote RMC with efforts that encompass enhancing the curriculum of health science programs and offering training to health professionals with its Health Sector Transformation Plan [19].

For the development and implementation of effective RMC interventions, it is essential to gain a better understanding of the etiology of mistreatment during childbirth [14]. Mistreatment during childbirth is a complex issue that not solely arises at a societal level (e.g. due to gender inequality) and at an organizational level (e.g. due to a lack of resources), but also at an individual level (e.g. due to care providers’ behavior towards women) [20]. Previous research has outlined some individual level risk factors among care providers; however, Ethiopia-specific research on this topic is scarce and it has been mostly conducted among working midwives [16,17]. Such research has in fact shown that care providers’ gender may constitute an important risk factor for mistreatment during childbirth in Ethiopia, with male gender being linked to greater competence and professionalism in the field of midwifery [17,21]. In a similar vein, research among Ethiopian healthcare providers has pointed towards more RMC provision among male midwives, which implies more patient abuse among female midwives, who form the majority (78%) of the Ethiopian midwifery workforce [16,22]. If female care providers are indeed more likely to mistreat women during childbirth, this constitutes an additional challenge to the use of maternal health services in certain ethnic groups, such as the Afar, who are reluctant to expose their reproductive health organs to male midwives due to cultural conventions [23].

Two factors that can possibly account for the above-mentioned gender differences are care providers’ self-esteem and stress. That is, lower self-esteem and more stress among females have been linked to unprofessionalism and aggression among healthcare staff [24–26]. Self-esteem can be defined as the overall evaluation of one’s worth as a person and stress can be defined as an emotional and physiological response to demands that exceed personal resources [27,28]. Healthcare staff in low-income settings often does not receive much acknowledgment and respect, which has been associated with low self-esteem [29]. Moreover, due to challenging working conditions that include frequent exposure to emotional needs of patients, as well as responsibility and task overload, stress levels in the healthcare branch are high [30,31]. A lack of basic resources in low-income settings further increases care providers’ stress levels [11].

Altogether, these findings lead to the two aims of this study, which were a) to generate a quantitative research scale that assesses mistreatment appraisal from a provider perspective, the Mistreatment Appraisal (MISAP) Scale, and b) to assess gender differences in mistreatment appraisal and the possible mediating roles of stress and self-esteem on the basis of this new scale. We hypothesize more positive appraisal of mistreatment during childbirth among female study participants. More stress and less self-esteem among females are thought to play a mediating role herein. Social desirability bias is likely to affect the assessment of mistreatment appraisal (i.e. causing lower mistreatment appraisal outcome scores). We decided to sample final-year midwifery students, instead of working midwives, in order to minimize the impact of social desirability bias. Final-year midwifery students have prior working experience, due to previously completing a practical internship. However, due to limited accountability, we believe students to answer questions more truthfully.

## Methods

### Design, setting and population

This study was conducted in two phases, related to the two aims of the study. In the first phase, we developed the MISAP Scale with suitable content validity. Therefore the following steps were needed:

- The identification of relevant forms of mistreatment during childbirth, using the typology of mistreatment by Bohren et al. [14];
- The development of a continuous scale of the appraisal of these distinguished types of mistreatment from a care provider perspective on the basis of a) scientific literature, and b) a review of the items by seven experts from the Ethiopian Ministry of Health, the Ethiopian Midwives Association, the Ethiopian Society of Obstetricians and Gynecologists, Jhpiego Ethiopia, Leiden University Medical Center and University Medical Center Groningen. These experts were all public health practitioners with master’s and/or PhD degrees in public health. They had 10 to 20 years of experience in public health and clinical practice. Experts were asked to evaluate questions’ relevance on a scale from 1 (*very irrelevant*) to 10 (*very relevant*), with relevance referring to ‘the quality of being appropriate for the context’. Clarity was assessed on a scale from 1 (*very unclear*) to 10 (*very clear*), which clarity referring to ‘the quality of being comprehensible’. Experts could also provide question-specific feedback by adding additional comments and suggestions. Relevance ratings were used to evaluate whether to retain or remove questions. The criterion for removing questions was a relevance rating of 5 or lower by at least two experts. Based on their comments, one item was excluded and all items were rephrased, which resulted in the 10-item MISAP Scale (S1 Appendix);
- The implementation of a pretest for which we sampled 11 final-year midwifery students from Menelik Health Science College (HSC) in November 2017, before the onset of the second phase. We concluded from this pretest that all questions were comprehensible.

In the second phase, final-year midwifery students from Gondar University, Bahir Dar University, Bahir Dar HSC, Hawassa HSC, Arsi University and Menelik HSC in Ethiopia were invited to participate in this study in November and December 2017. Local professional local data collectors were hired and interactions between foreign researchers and study participants were avoided in order to limit response bias. Data collectors provided instructions and ensured that students completed the questions individually, for which they were given as much time as needed. Sampling bias is very unlikely as students were approached during class and more than 99% of the approached students (392 of 393 students) agreed to participate. Two students were excluded from the analysis, due to not indicating a gender and completing an English version instead of an Ethiopian version of the questionnaire. The final study population consisted of 390 students (151 males, 239 females). The study protocol was reviewed and approved by the Johns Hopkins Bloomberg School of Public Health Institutional Review Board (IRB00008218). All students provided informed verbal group consent before participating.

Sample size calculations were based on expert ratings of our Ethiopian colleagues and conducted for proportions of two independent samples. We assumed a cut-off value of 55 for the MISAP Scale (i.e. the midpoint of theoretical total mean outcome scores), with values ≥55 being categorized as positive appraisal of mistreatment. In order to assess a difference between males and females of at least 15% (50 vs 65%), an alpha level of .05 and a power of .80 yielded a sample size of 362 (137 males and 225 females). We accounted for a sex ratio of 1.64, which was based on national student enrollment data that was previously collected by Jhpiego Ethiopia.

### Data collection

Students were invited to complete a paper-and-pen questionnaire, which included questions on background characteristics, self-esteem, stress and mistreatment appraisal. Previously translated Amharic versions of the Rosenberg Self-Esteem Scale and Perceived Stress Scale that showed acceptable degrees of internal consistency (α = .73 and α = .76, respectively) were used to assess self-esteem and stress [32,33]. Remaining questions were translated to Amharic and back-translated to English by two Ethiopian epidemiology master students from the University of Groningen, the Netherlands, before the implementation of the pretest. One student translated English questions to Amharic and the other student translated Amharic questions to English. Inconsistencies were subsequently discussed and adjusted.

### Variables

The *outcome variable* was mistreatment appraisal (α = .75) and it was assessed with the newly-developed, continuous 10-item MISAP Scale. Students were asked to rate actions that depict mistreatment on a scale ranging from 1 *(oppose strongly)* to 10 *(support strongly)*, yielding theoretical total mean outcome scores of 10 to 100, with higher scores depicting more positive mistreatment appraisal. We allowed 20% of the mistreatment appraisal items to be missing, which was the case in 6% (*N* = 25). When data were missing, weighted mean sum scores were calculated.

The *independent variable* was gender (male or female).

*Covariates* included institution (Gondar University, Bahir Dar University, Bahir Dar HSC, Hawassa HSC, Arsi University or Menelik HSC), ethnicity (Amhara, Oromo or other), place of origin (urban or rural), type of program (regular or extension, which is a program for individuals with prior working experience in the midwifery branch) and age (in years).

As *mediating variables*, self-esteem and stress were considered. Self-esteem (α = .61) was assessed with the 10-item Rosenberg Self-Esteem Scale, which ranges from 1 *(strongly agree)* to 4 *(strongly disagree)* and theoretically yields scores of 10 to 40, with higher scores indicating more self-esteem [34]. Due to ambiguous wording, one item *(I wish I could have more respect for myself)* was removed, which increased the scale’s internal consistency (α = .72), yielding theoretical scores of 9 to 36. Stress (α = .71) was measured with the 10-item Perceived Stress Scale, which ranges from 0 *(never)* to 4 *(very often)* and yields theoretical scores of 0 to 40, with higher scores depicting more stress [35]. We allowed 20% of the self-esteem and stress items to be missing, which was the case in 7% (*N* = 27) and 6% (*N* = 25), respectively. When data were missing, weighted mean sum scores were calculated.

In total, 1% of all values were missing (i.e. 35 values). To reduce the impact of missing data, we used multiple imputation to generate five datasets for our main analyses. Data imputation was conducted for place of origin (1 value), age (21 values), stress sum scores (7 values) and mistreatment appraisal sum scores (6 values). Imputed values were sampled from a predictive distribution based on the associations between all covariates, as well as all outcome and independent variables [36].

### Statistical analyses

Baseline characteristics of the study population were reported with descriptive statistics. Independent sample t-tests and chi-square (χ2) tests were used to assess gender differences at baseline.

The scale’s feasibility was determined by exploring missing values per item. Its reliability was assessed with the internal consistency coefficient, Cronbach’s alpha. To test the scale’s homogeneity, we calculated corrected item-total and inter-item correlations.

Univariable and multivariable linear regression analyses were applied to assess the association between gender and mistreatment appraisal, upon examining the assumptions of linearity [37]. Next mediation analyses were performed. Mediation analyses were conducted with the lavaan package version 0.6–3 in R statistical software version 3.5.1 for Windows. All other analyses were performed with SPSS statistical software version 25 for Windows. P-values < .05 were considered significant.

## Results

### Background characteristics

Table 1 displays background characteristics of the study population. Most students were enrolled at Gondar University (33%), identified as Orthodox (78%), indicated Amhara as their ethnicity (65%), had an urban origin (53%), attended a university (69%) and were enrolled in a regular study program (59%). Students were on average 24 years old and they had high self-esteem (*mean* 29.39, *SD* 4.27) and moderate stress levels (*mean* 15.22, *SD* 5.71). Significant gender differences were detected for four variables: females were more often enrolled at Gondar University (35% versus 31%), Hawassa HSC (12% versus 9%) and Menelik HSC (29% versus 9%), they originated more frequently from urban areas (65% versus 34%), attended a HSC (35% versus 25%) and/or followed an extension program (46% versus 33%).

**Table 1:** Background characteristics and mediating variables by gender.

Variable	Total (*N* = 390)	Female (*N* = 239)	Male (*N* = 151)	
	*Number (%)*	*Number (%)*	*Number (%)*	*p-value*
Institution				< .001
Gondar University	130 (33)	83 (35)	47 (31)	
Bahir Dar University	38 (10)	9 (4)	29 (19)	
Bahir Dar HSC	39 (10)	14 (6)	25 (16)	
Hawassa HSC	42 (11)	29 (12)	13 (9)	
Arsi University	59 (15)	35 (14)	24 (16)	
Menelik HSC	82 (21)	69 (29)	13 (9)	
Religion				.239
Orthodox	303 (78)	178 (74)	125 (83)	
Protestant	39 (10)	29 (12)	10 (7)	
Islam	42 (11)	28 (12)	14 (9)	
Other	6 (1)	4 (2)	2 (1)	
Ethnicity				.775
Amhara	254 (65)	154 (64)	100 (66)	
Oromo	66 (17)	43 (18)	23 (15)	
Other	70 (18)	42 (18)	28 (19)	
Place of origin				< .001
Urban	208 (53)	156 (65)	52 (34)	
Rural	181 (47)	82 (35)	99 (66)	
Missing	1 (0)	1 (0)	0 (0)	
Type of education				.047
University	269 (69)	155 (65)	113 (75)	
HSC	121 (31)	83 (35)	38 (25)	
Type of program				.009
Regular	229 (59)	128 (54)	101 (67)	
Extension	161 (41)	111 (46)	50 (33)	
	*mean (SD)* *Number (%)*	*mean (SD)* *Number (%)*	*mean (SD)* *Number (%)*	*p-value*
Age	23.59 (2.55)	23.47 (2.66)	23.78 (2.36)	.267
Missing	21 (5)	13 (5)	8 (5)	
Self-esteem	29.39 (4.27)	29.54 (4.17)	29.15 (4.44)	.380
Stress	15.22 (5.71)	15.41 (5.60)	14.92 (5.89)	.404

### Psychometric assessment of the MISAP Scale

In total, 59 values (2%) of all items of the MISAP Scale were missing. Table 2 shows that the percentage of missing values per item ranged from 1% to 3%. The internal consistency of MISAP Scale was acceptable (α = .75) and deleting any of the items would not have increased the scale’s internal consistency considerably. Corrected item-total correlations were acceptable, ranging between .24 and .56 [38]. Inter-item correlations were mostly acceptable, ranging from .07 to .63 (S1 Table) [39]. Item 7 was most frequently favored. Yet, this item also showed the lowest item-total, as well as inter-item correlations. This indicates that item 7 might measure a different construct than the other items. While all items of the MISAP Scale subject participants to circumstances that appear to justify mistreatment, item 7 might have been more readily appraised positively due to the hygiene rationale, which is frequently emphasized in the Ethiopian midwifery curriculum [40]. We did not remove item 7 from the analysis, as this would not have improved the internal consistency measure remarkably (.75≈.76). In fact, removing item 7 would have limited the scale’s scope, as all items of the MISAP Scale measure different forms of mistreatment.

**Table 2:** Item characteristics of the MISAP Scale in a cohort of Ethiopian midwifery students.

	Item									
	1	2	3	4	5	6	7	8	9	10
Missing values *Number* *(%)*	5 (1)	7 (2)	11 (3)	5 (1)	2 (1)	4 (1)	5 (1)	3 (1)	4 (1)	13 (3)
Cronbach's alpha if item deleted	.74	.72	.72	.72	.72	.71	.76	.71	.74	.72
Corrected item-total correlation	.35	.43	.42	.44	.46	.56	.24	.54	.33	.47

### The relationship between gender and positive mistreatment appraisal

**Fig 1:**
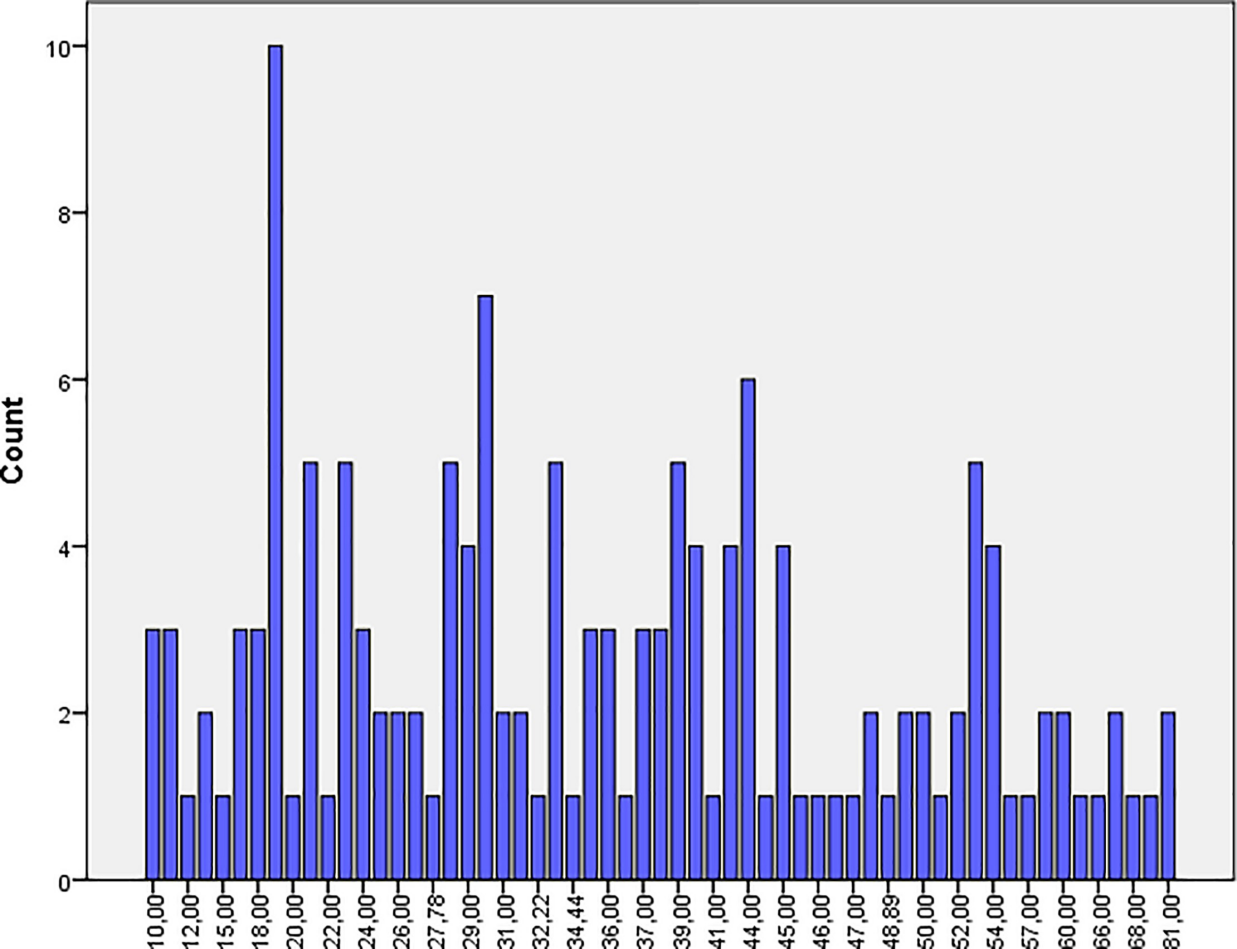
Outcome distribution of mistreatment appraisal for males.

**Fig 2:**
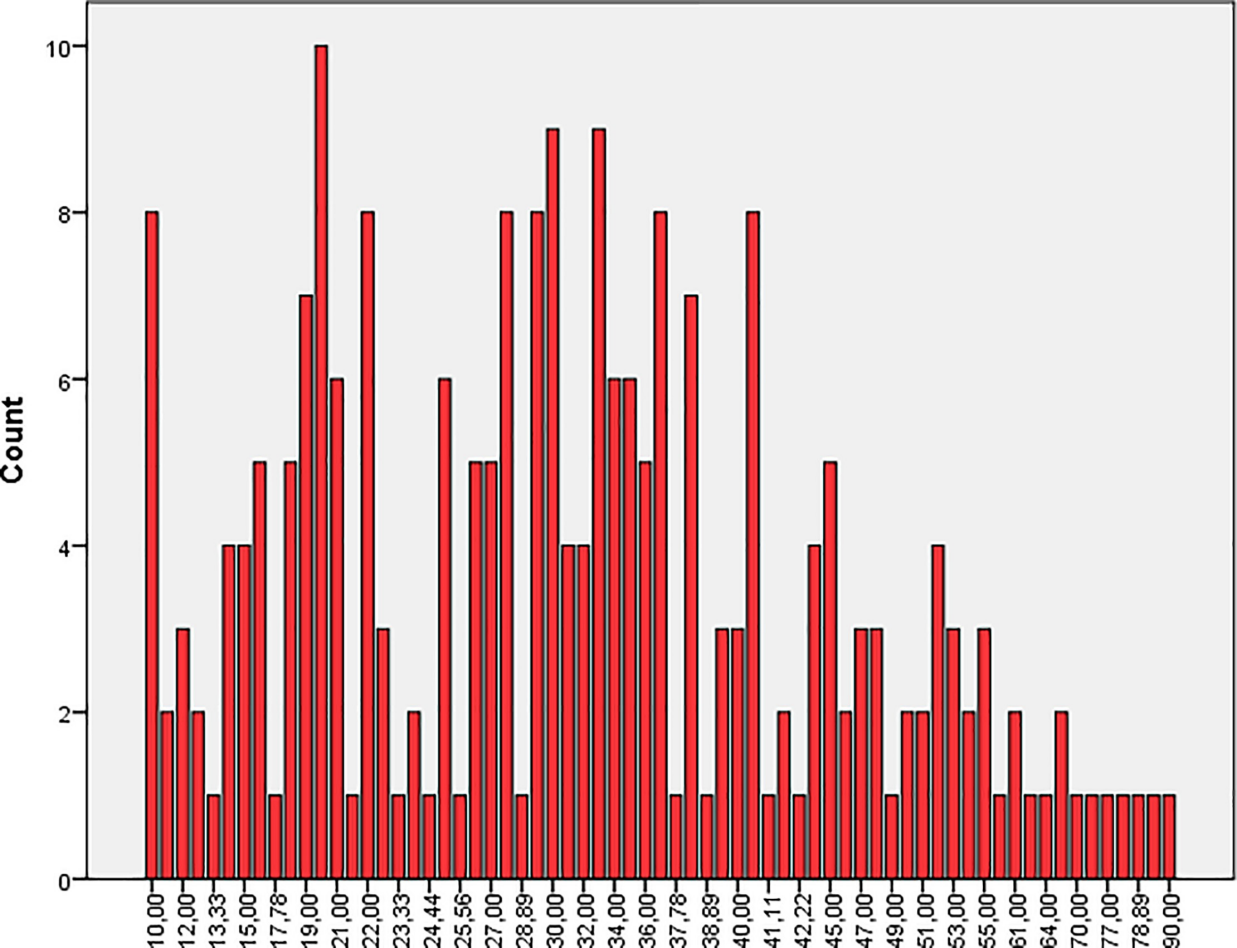
Outcome distribution of mistreatment appraisal for females.

**Fig 3:**
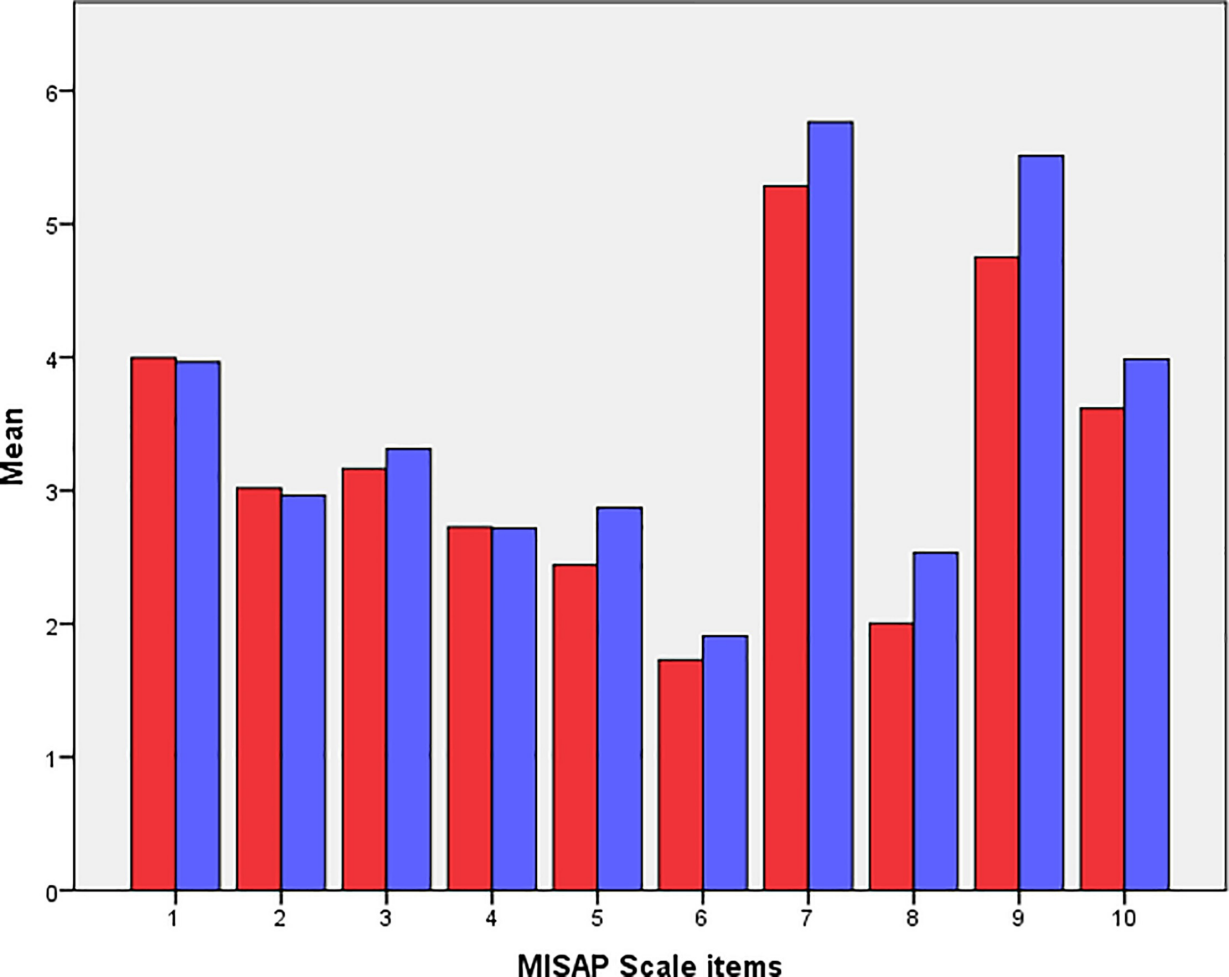
Mean scores for the different items of the MISAP Scale among males (blue) and females (red). Higher scores indicate more positive mistreatment appraisal, while lower scores indicate the opposite.

Students generally opposed mistreatment behavior (*mean* 33.93; *SD* 15.08). Male students appraised mistreatment more positively (*mean* 35.78; *SD* 15.24) than their female counterparts (*mean* 32.74; *SD* 14.88; Figs 1 and 2). There was some variability among male and female students with regard to the different items of MISAP Scale (Fig 3). Item 7 was favored most frequently.

When applying univariable linear regression analysis, no significant difference between male and female midwifery students regarding mistreatment appraisal was observed (*B* = 3.084, 95% CI [-.005, 6.173]). Seven confounders (institution, ethnicity, place of origin, type of program, age, self-esteem, stress) were identified and adjusted for in a multivariable linear regression analysis. The association between gender mistreatment appraisal remained insignificant in this model (*B* = 1.867, 95% CI [-1.472, 5.205]).

**Fig 4:**
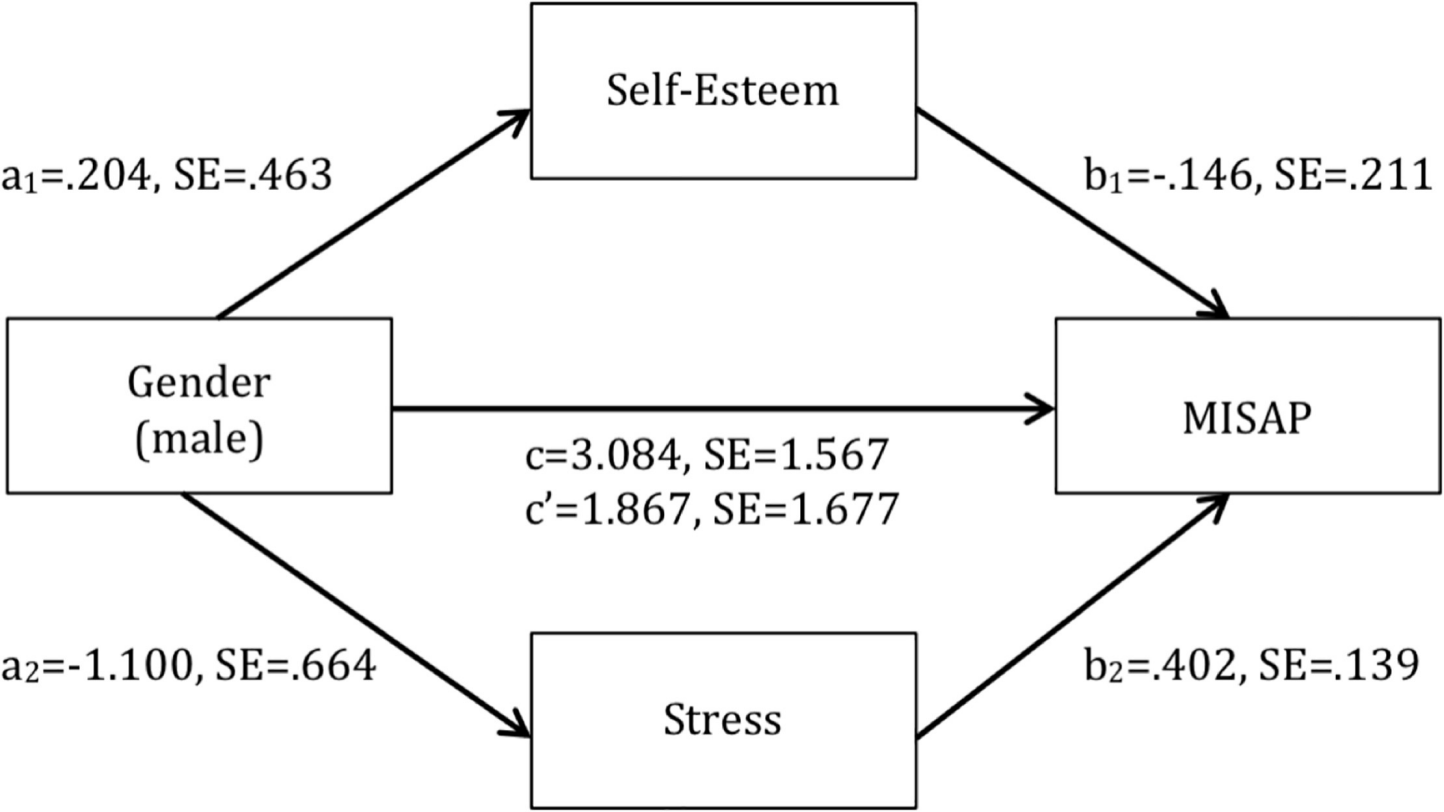
Unstandardized regression coefficients and standard errors for the relationship between gender and mistreatment appraisal as mediated by self-esteem and stress and adjusted for confounding of institution, ethnicity, place of origin, type of program and age. *p < .05.

The indirect effect of gender on mistreatment appraisal via self-esteem and stress was insignificant, *a*_*1*_*b*_*1*_ = -.030, *p* = .677 and *a*_*2*_*b*_*2*_ = -.443, *p* = .186, respectively (Fig 4).

## Discussion

Our study showed that we succeeded in developing a quantitative research scale, the MISAP Scale, that measures mistreatment appraisal from a provider perspective with acceptable psychometric properties, and that female Ethiopian midwifery students did not have more positive mistreatment appraisal scores than their male counterparts. Self-esteem and stress did not mediate the effect of gender on mistreatment appraisal.

The MISAP Scale showed acceptable psychometric properties: good feasibility characteristics, an acceptable internal consistency reliability, acceptable corrected item-total correlations and mostly acceptable inter-item correlations [38,39]. Content validity was ensured through employing relevant literature and conducting an expert review.

We did not observe a significant association between gender and mistreatment appraisal. This is not in line with previous research findings in the Ethiopian context that have linked male gender to more RMC provision, as well as competence and professionalism in the field of midwifery [17,21,41]. A factor that may account for this unexpected finding is that we may have assessed and operationalized mistreatment during childbirth in a way that is not consistent with previous studies: We utilized a quantitative research scale to assess individuals’ mistreatment appraisal in circumstances that appear to justify mistreatment, while previous research observed maternity care provision in health facilities and used in-depth interview techniques [16,17]. In line with the notion that attitudes not necessarily equate to behavior, low mistreatment appraisal scores might not necessarily equate to RMC provision [42]. In this study both males and females opposed mistreatment, yet in reality females might follow this maxim less frequently [43]. Validation studies are needed to assess if the MISAP Scale measures the inclination of health providers to mistreat women during childbirth. A distinct feature of this study is that we captured the perspective of midwifery students, while previous research on gender differences in maternity care provision captured the perspective of healthcare professionals and patients. Accordingly, some patients in an Ethiopian sample indicated a preference for treatment by male midwives [17]. However, this does not necessarily have to stem from an actual performance difference between male and female healthcare personnel. Patients might hold different expectations for male and female health professionals, which may bias their evaluations despite equal provider performance [44].

Self-esteem and stress were not found to mediate the effect of gender on mistreatment appraisal. This also does not conform to previous research that has pointed towards lower self-esteem and more stress among females, which in turn has been linked to unprofessionalism and aggression among healthcare staff [24–26]. There are various explanations that may account for this finding. First, gender initiatives in Ethiopia have received more and more support throughout the last years [45]. Gender initiatives often target students in particular, for example via girls’ clubs and university associations. This might have led to a reduction of gender-bound stressors and an increase of self-esteem for young female individuals, which can account for similar mistreatment appraisal ratings among male and female study participants. Second, most midwifery students did not have children, while this will most likely change throughout early and middle adulthood. Women in developing countries often need to fulfill not only economic roles but also reproductive and community management tasks, without monetary compensation or social benefits [46]. While most female midwives are exposed to both demanding jobs and gender-bound social demands (e.g. being a good mother and wife, managing the household), this might not yet hold for most female midwifery students. Thus, fewer responsibilities and adequate fulfillment of social obligations among female midwifery students, as compared to female midwives, may imply higher self-esteem and reduced stress levels [47,48]. In turn, this offers an explanation for the finding that associations between gender and self-esteem, gender and stress, as well as gender and mistreatment appraisal were insignificant. Third, research that compared the concept of self-esteem in 53 nations indicated low internal consistency for the Rosenberg Self-Esteem Scale in Ethiopia (α = .64), especially for one question (*I wish I could have more respect for myself*; α = .33), which was also removed in our analysis [49]. As Ethiopians did not answer comparable to different questions, self-esteem in its global form might be a less tangible concept in the Ethiopian setting [50,51].

An important strength of this study is the data quality and its quantitative design, which allowed us to use standardized questionnaires to get an objective understanding of the issue at hand. According to first psychometric assessments, the MISAP Scale appears to be a feasible and reliable instrument, and its content validity was ensured through employing relevant literature and conducting an expert review. The study also has a number of limitations. The most important limitation is that even though first psychometric assessments of the MISAP Scale were positive, it possibly did not successfully differentiate between those who appraise mistreatment positively versus those who appraise mistreatment negatively. Moreover, it should be noted that the MISAP Scale was primarily developed using literature. In line with previous research, we decided to develop items by first conducting a literature review and then assessing the scale’s content validity with the help of an expert panel [52]. Care should be applied when generalizing findings, as we sampled students from four of Ethiopia’s 11 regions (Addis Ababa, Amhara Region, Oromia Region and Southern Nations, Nationalities, and Peoples' Region), which are very diverse in terms of ethnicity [53]. Thus, our sample might not be fully representative of the entire Ethiopian population. Moreover, due to the cross-sectional set-up of this study, no causal inferences can be drawn. Despite not revealing the exact purpose of this study and emphasizing anonymity, social desirability bias is likely to affect the assessment of mistreatment appraisal. We assume that sampling final-year midwifery students, as opposed to working midwives, allowed us to minimize social desirability bias due to limited accountability in this population.

There is no doubt that quality care provision, which encompasses RMC, is needed in order to improve maternal and neonatal health outcomes in Ethiopia and beyond [54]. Utilizing the quantitative MISAP Scale can contribute towards the development of more effective RMC interventions in the future, as it offers more generalizable insights than previous qualitative studies [14]. The present study showed that 1) we were able to develop a feasible and reliable instrument, the MISAP Scale, measuring mistreatment appraisal during childbirth and 2) that positive mistreatment appraisal, measured with this quantitative scale, is opposed by most Ethiopian midwifery students, both males and females. Yet, there were still few students that frequently appraised mistreatment positively. As any form of mistreatment behavior can yield adverse health effects among mothers and their newborns, results of this study still underline the need of promoting RMC among Ethiopian midwifery students. Our findings do not offer support for the implementation of gender-specific RMC interventions in Ethiopia, they might however point towards the success of previous gender initiatives.

## Supporting information

S1 Appendix MISAP Scale. (DOCX)

S2 Appendix English questionnaire. (DOCX)

S3 Appendix Amharic questionnaire. (PDF)

S4 Appendix Original data. (SAV)

S1 Table Inter-item correlations of the MISAP Scale in a cohort of Ethiopian midwifery students. (DOCX)
