# Supplementary material for: Development and use of a scale to assess gender differences in appraisal of mistreatment during childbirth among Ethiopian midwifery students
Source: PLoS One. 2020 Jan 16;15(1):e0227958. doi: 10.1371/journal.pone.0227958 (PMC6964878; doi:10.1371/journal.pone.0227958)
Supplement: S2 Appendix — (DOCX) [file pone.0227958.s002.docx]

**Gender Differences in Acceptance of Mistreatment of Women during Childbirth in Ethiopia**

**PART I. Background information**

Firstly, we would firstly like to ask you to provide some background details. Mark the correct answer by making a cross (x) accordingly.

101. Age: ....................................

102. Gender:

- Male
- Female

103. Religion:

- Orthodox
- Protestant
- Islam
- Other, namely: ....................................

104. Ethnic background:

- Oromo
- Amhara
- Somali
- Tigray
- Other, namely: ....................................

105. Place of origin:

- Urban
- Rural

106. Do you have children (If yes, indicate how many)?

- No
- Yes, I have .................................... children

107. Type of education:

- University
- Health Science College

108. Educational stage:

- Third year student or below
- Fourth year student

109. How many deliveries did you attend during your education so far?

....................................

**PART II.**

Life as a young adult can be quite demanding. The questions in this scale ask you about your feelings and thoughts during the last month. In each case, you will be asked to indicate by cycling how often you felt or thought a certain way.

|  |  |  | | | | |
| --- | --- | --- | --- | --- | --- | --- |
| # | In the last month, how often have you been/felt: | **0 = Never** | **1 = Almost Never** | **2 = Sometimes** | **3 = Fairly Often** | **4 = Very Often** |
| 201 | Upset because of something that happened unexpectedly? | 0 | 1 | 2 | 3 | 4 |
| 202 | Felt that you were unable to control the important things in your life? | 0 | 1 | 2 | 3 | 4 |
| 203 | Nervous and “stressed? | 0 | 1 | 2 | 3 | 4 |
| 204 | Confident about your ability to handle your personal problems? | 0 | 1 | 2 | 3 | 4 |
| 205 | Things were going your way? | 0 | 1 | 2 | 3 | 4 |
| 206 | Found that you could not cope with all the things that you had to do? | 0 | 1 | 2 | 3 | 4 |
| 207 | Able to control irritations in your life? | 0 | 1 | 2 | 3 | 4 |
| 208 | You were on top of things? | 0 | 1 | 2 | 3 | 4 |
| 209 | Angered because of things that were outside of your control? | 0 | 1 | 2 | 3 | 4 |
| 210 | Difficulties were piling up so high that you could not overcome them? | 0 | 1 | 2 | 3 | 4 |

**PART III.**

| # | Below is a list of statements dealing with your general feelings about yourself. Please indicate how strongly you agree or disagree with each statement. | Responses | | | |
| --- | --- | --- | --- | --- | --- |
|  |  | 1=  Strongly Disagree | 2=  Disagree | 3=  Agree | 4=  Strongly Agree |
| 301 | On the whole, I am satisfied with myself | 1 | 2 | 3 | 4 |
| 302 | At times I think I am no good at all | 1 | 2 | 3 | 4 |
| 303 | I feel that I have a number of good qualities | 1 | 2 | 3 | 4 |
| 304 | I am able to do things as well as most other people | 1 | 2 | 3 | 4 |
| 305 | I feel I do not have much to be proud of | 1 | 2 | 3 | 4 |
| 306 | I certainly feel useless at times | 1 | 2 | 3 | 4 |
| 307 | I feel that I'm a person of worth, at least on an equal plane with others | 1 | 2 | 3 | 4 |
| 308 | I wish I could have more respect for myself | 1 | 2 | 3 | 4 |
| 309 | All in all, I am inclined to feel that I am a failure | 1 | 2 | 3 | 4 |
| 310 | I take a positive attitude toward myself | 1 | 2 | 3 | 4 |

**PART IV.**

Midwives often have to work under very challenging conditions. Below, multiple scenarios with associated actions are presented. On a scale from 1 to 10, with 1 = very much disapprove of and 10 = very much approve of, please rate the actions by making a cross (x) accordingly.

401. A woman is constantly closing her legs during the second stage of labor. A midwife tells her that she should not do that, as he/she believes that the baby will not deliver, yet the woman continues to move her legs together. Each time the woman closes her legs, the midwife slaps her legs and forces them apart again.

How do you judge the action of the midwife?


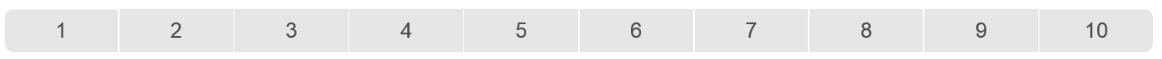


very much disapprove of very much approve of

402. An episiotomy (surgical cut at opening of vagina) is performed at an obstetric health facility due to fetal distress. The woman is illiterate and comes from a rural area. The midwife believes that the woman will not understand the medical procedure and that offering explanations would be a waste of time. In order to quicken childbirth, the midwife carries out the episiotomy without any explanation and getting the woman’s permission.

How do you judge the action of the midwife?


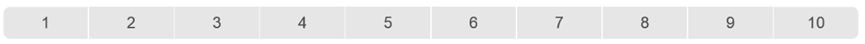


very much disapprove of very much approve of

403. On a busy day, the admission room of a district hospital is overcrowded with many emergency cases. During the admission of one of the cases – a woman in advanced labor who has vaginal bleeding and is very anxious – the midwife does not offer explanations about what he/she is doing or any findings on the procedures to the woman and her sister who accompanied her.

How do you judge the action of the midwife?


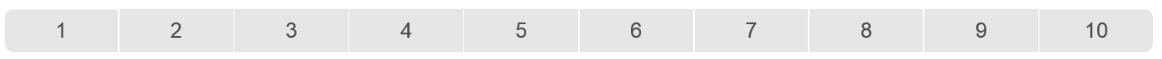


very much disapprove of very much approve of

404. A midwife is caring for a woman in labor who is HIV positive. In order to limit the risk of infection, the midwife believes she needs to tell the woman’s HIV status to a colleague, who works at the outpatient department and who is not directly involved in the care of that woman during lunch break in the cafeteria.

How do you judge the action of the midwife?


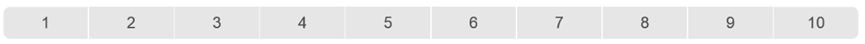


very much disapprove of very much approve of

405. A 16-year-old girl is visiting a health facility for safe abortion care at 10 weeks in a district hospital. Before the procedure is carried out, the girl is asked whether she has ever had an abortion. The girl indicates that she has had two abortions within the last two years, due to poor contraceptive use. During the procedure, she is weeping loudly, despite receiving painkillers. A midwife tells her in a strict tone: ‘If you find the procedure painful, stop sleeping with a man at every opportunity you get!’

How do you judge the action of the midwife?


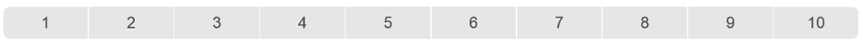


very much disapprove of very much approve of

406. A midwife that works at a health center provides all aspects of maternity care services. There are many women waiting for their turn. Some of the clients are from the same area as the midwife. Some women who speak a different language arrived at the facility earlier, yet the midwife attends to the women from her area first.

How do you judge the action of the midwife?


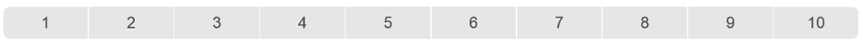


very much disapprove of very much approve of

407. A midwife believes that if companions are allowed in the delivery room, the cleanliness of the room will be compromised, which poses a risk to the health of newborns. Moreover, the midwife thinks that companions reduce patients’ privacy, given the fact that multiple women give birth in the same room. A woman asks if her mother can be present during her delivery but the midwife denies this request for the above-mentioned reasons.

How do you judge the action of the midwife?


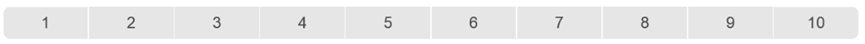


very much disapprove of very much approve of

408. A mother with postpartum bleeding arrives at a health facility following a home birth. She has lost about 400 ml of blood within the first 24 hours following childbirth and is unwell. She waits two hours for a midwife that works alone to finish attending to two births. After the deliveries, the midwife is exhausted and wants to go home as the scheduled shift is over. Therefore, the midwife decides to refer the woman to another health facility, which is one hour away.

How do you judge the action of the midwife?


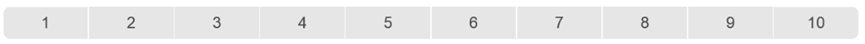


very much disapprove of very much approve of

409. A woman wants to give birth in a kneeling position, however a midwife believes that the bed is not suited for that position. Hence, the midwife forces the woman to give birth in a lying position.

How do you judge the action of the midwife?


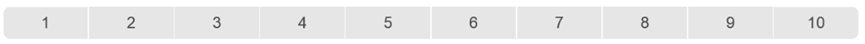


very much disapprove of very much approve of

410. A midwife attends to a woman that came for delivery services to a district hospital. Other women are also being attended to in the same room. The woman is shouting and crying and others feel disturbed. The healthcare personnel are finding it hard to concentrate when carrying out routine tasks. A midwife tells the woman to be quiet, yet the woman continues to make a lot of noise. Eventually, the midwife yells at the woman to be quiet using very harsh language.

How do you judge the action of the midwife?


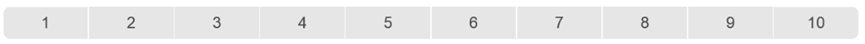


very much disapprove of very much approve of

**PART V.**

We would like to ask you some questions about all of the deliveries you have watched or participated in as part of your school.

|  | Do you remember the provider (nurse, midwife, physician) … | 1= Never | 2= Rarely | 3= Sometimes | 4=  Most of the time |
| --- | --- | --- | --- | --- | --- |
| 501 | Holding the laboring woman’s hand? | 1 | 2 | 3 | 4 |
| 502 | Slapping the laboring woman to encourage her to push? | 1 | 2 | 3 | 4 |
| 503 | Asking family members to participate in the delivery process? | 1 | 2 | 3 | 4 |
| 504 | Telling the family members to leave? | 1 | 2 | 3 | 4 |
| 505 | Shouting or yelling at the laboring woman to encourage her to push? | 1 | 2 | 3 | 4 |
| 506 | Telling the laboring woman that she was doing a good job? | 1 | 2 | 3 | 4 |
| 507 | Asking the laboring woman to stop making so much noise? | 1 | 2 | 3 | 4 |
| 508 | Leaving the laboring woman alone for long periods of time? | 1 | 2 | 3 | 4 |
| 509 | Asking the woman if she needed anything? | 1 | 2 | 3 | 4 |
| 510 | Detaining a laboring women or her baby who could not pay for her care? | 1 | 2 | 3 | 4 |
| 511 | Speaking disrespectfully to the laboring woman or her family? | 1 | 2 | 3 | 4 |
| 512 | Explaining to the laboring woman what was going to happen to her? | 1 | 2 | 3 | 4 |
| 513 | Asking the laboring woman if she had any questions? | 1 | 2 | 3 | 4 |
| 514 | Treating wealthy women better than poor women? | 1 | 2 | 3 | 4 |
| 515 | Treating educated women better than women who are not as educated? | 1 | 2 | 3 | 4 |
| 516 | Asking the laboring woman for her permission before examining her? | 1 | 2 | 3 | 4 |
| 517 | Scolding the laboring woman if she did not bring the right birth supplies? | 1 | 2 | 3 | 4 |
