# Supplementary material for: Development and use of a scale to assess gender differences in appraisal of mistreatment during childbirth among Ethiopian midwifery students
Source: PLoS One. 2020 Jan 16;15(1):e0227958. doi: 10.1371/journal.pone.0227958 (PMC6964878; doi:10.1371/journal.pone.0227958)
Supplement: S3 Appendix — (PDF) [file pone.0227958.s003.pdf]

Gender Differences in Acceptance of Mistreatment of Women during Childbirth in Ethiopia

ክፍል 1 በመጀመሪያ የግል መግለጫዎችን እንድትሞሉ እንጠይቃለን። ትክክለኛውን መልስ ለማሳየት X ምልክትን ይጠቀሙ።

101. እድሜ: ____________________

102. ጾታ

☐ ወንድ

☐ ሴት

103. ሐይማኖት

☐ ኦርቶዶክስ
☐ ፕሮቴስታንት
☐ ሙስሊም

☐ ሌላ፣በስም ይገልጽ________________________________

104. ብሄር

☐ ኦሮሞ
☐ አማራ
☐ ሱማሌ
☐ ትግሬ
☐ ሌላ፣በስም ይገልጽ________________________________

105. የቀድሞ መኖሪያ

☐ ከተማ
☐ ገጠር

106. ልጆች አሉህ/ሽ (መልሱ አዎ ከሆነ ስንት)

☐ አይደለም
☐ አዎ፣ ________ልጆች አሉኝ

107. የትምህርት ተቋም

☐ ዩኒቨርስቲ
☐ ጤና ሳይንስ ኮሌጅ

108. የትምህርት አመት

☐ ሦሥተኛ አመት ተማሪ ወይም ከዚያ በታች
☐ አራተኛ አመት ተማሪ

109. እስከ አሁን በነበረህ/ሽ የትምህርት ቆይታ ስንት የወሊድ አገልግሎቶች ላይ ተሳትፈሀል/ሻል?

____

ክፍል 2

በወጣትነት ህይወት ከባድ ሊሆን ይችላል። በዚህ መለኪያ ያሉት ጥያቄዎች ባለፈው ወር ውስጥ የነበራችሁን ስሜት እና ሀሳብ ይጠይቃሉ። በእያንዳንዱ ሁኔታ ምን ያህል ጊዜ በዚህ መልኩ እንደተሰማህ/ሽ እና እንዳሰብክ/ሽ እንድታሳዩ ትጠየቃላችሁ።

#	ባለፈው ወር ውስጥ ምን ያህል ጊዜ ቀጥሎ የተዘረዘሩት ስሜቶች ተሰምቶህል/ሻል	0= ምንም ጊዜ አልነበረም	1= በጣም አልፎ አልፎ	2= አልፎ አልፎ	3= አብዛኛውን ጊዜ	4= ሁልጊዜ
201	ሳይጠበቅ ድንገት በተከሰተ ነገር ምክንያት ምን ያህል ጊዜ ተረብሸው ወይም አዝነው ያውቁ ነበር?	0	1	2	3	4
202	የዕለት ተዕለት የህይወት እንቅስቃሴዎን መቆጣጠር የማይችሉ አይነት ስሜት ምን ያህል ጊዜ ተሰምቶዎ ነበር?	0	1	2	3	4
203	የአዕምሮ ጭንቀት ወይም ውጥረት ምን ያህል ጊዜ ያጠቃዎ ነበር?	0	1	2	3	4
204	ችግሮች ባጋጠሙዎ ጊዜ ችግሮችን የመፍታት ወይም የመወጣት ችሎታዎን በተመለከተ ምን ያህል ጊዜ በራስ የመተማመን ስሜት ይሰማዎ ነበር?	0	1	2	3	4
205	ነገሮች ሁሉ እርስዎ እንደሚፈልጉት እየሄዱልዎ ወይም እየተከናወኑልዎ እነደሆነ አይነት ስሜት ምን ያህል ጊዜ ይሰማዎ ነበር?	0	1	2	3	4
206	እርስዎ ማንኛውንም ማከናወን ያለብዎን ነገር በብቃት መወጣት የማይችሉ ሰው እንደሆኑ ምን ያህል ጊዜ ይሰማዎ ነበር?	0	1	2	3	4
207	ያጋጠሙዎትን የህይዎት ውጣ ውረዶች ወይም ፈታኝ ገጠመኞች ምን ያህል ጊዜ በብቃት መወጣት ችዬ ነበር ብለው ያምናሉ?	0	1	2	3	4
208	8 የሚያጋጥሙዎት ችግሮች ሁሉ በርስዎ ቁጥጥር ሥር እንደሆኑ ዓይነት ስሜት ምን ያህል ጊዜ ተሰምቶዎት ነበር?	0	1	2	3	4
209	ከቁጥጥርዎ ውጪ በሆኑ ነገሮች ምክንያት ምን ያህል ጊዜ ተበሳጭተው ያውቁ ነበር?	0	1	2	3	4
210	የተለያዩ ችግሮች ባጋጠሙዎት ጊዜ፡ ችግሮቹ ከአቅምዎ በላይ እንደሆኑና እርስዎ ችግሮችን መወጣት የማይችሉ እነደሆኑ ምን ያህል ጊዜ ይሰማዎ ነበር?	0	1	2	3	4

ክፍል 3

#	*መመሪያ: ከ□ች ከተ□ረ□ሩት □□ቄዎች ስለራስሽ □ለሽን ስሜት □ቶል□ል □ምትይውን ምር□ በ□ሞና በማንበብ አክብብ/ቢ::*	**1**<br>*□ ራሽ አልስማማም*	**2**<br>*አልስማማም*	**3**<br>*□ስማማሁ*	**4**<br>*በ□ም □ስማማለሁ*
301	ቢያንስ ከሌሎች ሰዎች ጋር ተመጣጣኝ በሆነ መልኩ ዋጋ ያለኝ ሠው □ንደሆንኩ □ሰማኛል::	1	2	3	4
302	□ኔ መልካም የሆኑ ባህሪያቶች □ንዳሉኝ □ሰማኛል::	1	2	3	4
303	ባ□ቃላ□ □ኔ □ማ□ሳካልኝ ሰ□ □ንደሆንኩ □ሰማኛል::	1	2	3	4
304	ሌሎች ብዙዎች ሠዎች የሚሠሩትን ያህል መስራት □ችላለሁ::	1	2	3	4
305	□ኔ ያን ያህል የምኮራበት ነገር □ንደሌለኝ □ሰማኛል::	1	2	3	4
306	□ኔ ስለራሴ ያለኝ አመለካከት አዎን□ዊ ነው::	1	2	3	4
307	በአጠቃላይ □ኔ በራሴ ሁኔ□ በ□ም □ስተኛ ነኝ::	1	2	3	4
308	□ኔ ስለራሴ የበለጠ ክብር □ንዲኖረኝ □መኛለሁ::	1	2	3	4
309	□ኔ በ□ርግጠኝነት □ማረባ ሰ□ □ንደሆንኩ □ሰማኛል::	1	2	3	4
310	አንዳንዴ ስለራሴ ሳስብ በሀሉም ነገር ጥሩ □ንዳልሆንኩ □ሰማኛል::	1	2	3	4

ክፍል 4

ሚድዋይፎች በብዛት በፈታኝ ሁኔታዎች ለመስራት ይገደዳሉ። ከዚህ በታች የተለያዩ ክስተቶች ከነእርምጃዎቻቸው ቀርበዋል። እነዚህን እርምጃዎች ከ 1 እስከ 10 ባለ ነጥብ ማለትም ከ 1 በጣም እቃወማለሁ እስከ 10 በጣም ደግፋለሁ መለኪያ ላይ የ X ምልክት በማስቀመጥ ገምግሞቸው።

401. አንዲት በወሊድ ላይ ያልች ሴት ሁለተኛ ደረጃ ላይ የደረሰ ምጥ ላይ ሆና ያለማቋረጥ ጭኗን ዘግታለች። ሚድዋይፉ/ዋ ይህንን እንዳታደርግ ይነግራታል/ትነግራታለች ምክንያቱም የወሊድ ሂደቱን አስቸጋሪ ያደርጋል ተብሎ ስለሚታሰብ። ነገርግን ሴትዮዋ አሁንም በመቀጠል ጭኗን ዘግታለች። ጭኗን በምትዘጋበት ጊዜ አዋላጅ ነርስ እግሯን መታት/ቻት ጭኗን እንድትከፍት አስገደዳት/ቻት።
ይህንን የአዋላጅ ነርስ እርምጃ እንዴት ትገመግመዋለህ/ሚዋለሽ?

1	2	3	4	5	6	7	8	9	10

በጣም እቃወማለሁ　　　　　　　　　　　　　　　　　　　　　　　　　　　　በጣም ደግፋለሁ

402. በወሊድ ተቋም ውስጥ በህጻን መታፈን ምክንያት በብልት መግቢያ አካባቢ ቀዶ ጥገና (Episiotomy) ይካሄዳል። ከገጠር አካባቢ የመጣች ያልተማረች እናት ነበረች። አዋላጅ ነርሱ/ሷ በምጥ ለይ ያለችው እናት የህክምናውን ሂደት አትረዳም እና ይህንን ማስረዳት ጊዜ ማባከን ነው የሚል እምነት አለው/ላት። የወሊድ ሂደቱ እንዲፋጠን አዋላጅ ነርሱ/ሷ ያለ ማብራሪያ እና ከእናቷ ፍቃድ ውጪ በብልት መግቢያ አካባቢ ቀዶ ጥገና (Episiotomy) ተከናውኗል።

ይህንን የአዋላጅ ነርስ እርምጃ እንዴት ትገመግመዋለህ/ሚዋለሽ?

1	2	3	4	5	6	7	8	9	10

በጣም እቃወማለሁ　　　　　　　　　　　　　　　　　　　　　　　　　　　　በጣም ደግፋለሁ

403. ስራ በበዛበት ቀን የሆስፒታል ታካሚ መቀበያ በድንገተኛ ታካሚዎች ተጨናንቋል። አንዲት ታካሚን በመቀበያ ወቅት ታካሚዋ የገፋ ምጥ ለይ ሆና የማህጸን ደም መፍሰስ አላት እና ተጨንቃለች። አዋላጅ ነርሱ/ሷ ምን እንደምያደርግ/ታደርግ ወይንም ስለ ምርመራ ውጤቷ ለታካሚዋም ሆነ አብራት ላለች እህቷ አላብራራም/ችም።

ይህንን የአዋላጅ ነርስ እርምጃ እንዴት ትገመግመዋለህ/ሚዋለሽ?

1	2	3	4	5	6	7	8	9	10

በጣም እቃወማለሁ　　　　　　　　　　　　　　　　　　　　　　　　　　　　በጣም ደግፋለሁ

404. አዋላጅ ነርሱ/ሷ በምጥ ለይ ያለች የHIV ቫይረስ በደሟ ውስጥ ያለባት እናትን እየተንከባከበ/ች ነው። በቫይረስ የመጋለጥ እድልን ለመቀነስ አዋላጅ ነርሱ/ሷ በምጥ ለይ ያለችው እናት የHIV ቫይረስ በደሟ ውስጥ እንዳለ በተመላላሽ ታካሚ ክፍል ላለ እና በእናትዋ ህክምና ለይ በቀጥታ ለማይሳተፉ የስራ ባልደረባ በምሳ እረፍት ለይ ምግብ ቤት ውስጥ ይነግራል/ትነግራለች።

ይህንን የአዋላጅ ነርስ እርምጃ እንዴት ትገመግመዋለህ/ሚዋለሽ?

1	2	3	4	5	6	7	8	9	10

በጣም እቃወማለሁ　　　　　　　　　　　　　　　　　　　　　　　　　　　　በጣም ደግፋለሁ

405. የ16 አመት ልጅ 10 ሳምንት እርግዝና ለማስወረድ ወደ ጤና ተቋም መጥታለች። ከውርጃ ሂደት በፊት ታካሚዋ ከዚህ በፊት ውርጃ አድርጋ የምታውቅ ከሆነ ተጠየቀች። ታካሚዋ ከዚህ በፊት ሁለት ውርጃ ባለፉት ሁለትአመት ውስጥ በወሊድ መከላከያ ችግር ምክንያት አድርጋ የምታውቅ መሆኑን ገለጸች። በውርጃ ሂደት ለይ የህመም ማስታገሻ ብትወስድም በጩህት እያለቀሰች ነው። አዋላጅ ነርሱ/ሷ በቁጣ "ይሄ ህክምና ካሳመመሽ ባገኘሽው አጋጣሚ ከወንድ ጋር መተኛትሽን አቀኅሚ" አላት/ቻት።

ይህንን የአዋላጅ ነርስ እርምጃ እንዴት ትገመግመዋለህ/ሚዋለሽ?

1	2	3	4	5	6	7	8	9	10

በጣም እቃወማለሁ በጣም ደግፉለሁ

406. በጤና ተቋም ውስጥ የአዋላጅ ነርሶች የተለያየ የጤና አገልግሎቶች ይሰጣሉ። ብዙ ሴቶች ተራቸውን በመጠበቅ ለይ ናቸው። አንዳንድ ታካሚዎች የአዋላጅ ነርሱ/ሷ ከመጣችበት አካባቢ የመጡ ናቸው። ቀድመው የመጡ የተለየ ቋንቋ የሚናገሩ አንዳንድ ሴቶች ቢኖሩም አዋላጅ ነርሱ/ሷ ከመጣችበት አካባቢ የመጡ ታካሚዎችን በቅድሚያ ታከሙ።

ይህንን የአዋላጅ ነርስ እርምጃ እንዴት ትገመግመዋለህ/ሚዋለሽ?

1	2	3	4	5	6	7	8	9	10

በጣም እቃወማለሁ በጣም ደግፉለሁ

407. አዋላጅ ነርሱ/ሷ አስታማሚ ማዋለጃ ክፍል ውስጥ ቢገባ የክፍሉ ጽዳት እንደማይጠበቅ እና ይሄ ደሞ ህጻኑን ለጤና ችግር ያጋልጣል ብሎ/ላ ያምናል/ታምናለች። በተጨማሪ አዋላጅ ነርሱ/ሷ ብዙ እናቶች በአንድ ክፍል ለይ ስለሚወልዱ የታካሚን ግላዊነትን ይቀንሳል ብሎ/ላ ያምናል/ታምናለች። አንድ ታካሚ በወሊድ ሰአት እናቷ አብራት እንድትሆን ብትጠይቅም አዋላጅ ነርሱ/ሷ ከላይ በተዘረዘሩት ምክንያቶች መሰረት ከለከላት/ለቻት።

ይህንን የአዋላጅ ነርስ እርምጃ እንዴት ትገመግመዋለህ/ሚዋለሽ?

1	2	3	4	5	6	7	8	9	10

በጣም እቃወማለሁ በጣም ደግፉለሁ

408. አንድ እናት ቤት ውስጥ ከወለደች በኌላ ከወሊድ በኋላ በሚያጋጥም ደም መፍሰስ ምክንያት ወደ ጤና ተቋም መጣች ከወለደች በኌላ ባሉት 24 ሰአት ውስጥ 400ml የሚሆን ደም ፈሷታል እናም አ ሟታል። ታካሚዋ ሌላ ሁለት ወሊዶችን ሲረዳ/ስትረዳ የነበረ/ች አዋላጅ ነርስ ለሁለት ሰአት ጠበቀች። ከወሊዶቹ በኌላ አዋላጅ ነርሱ/ሷ ስለደከመው/ማት እና የስራ ሰአት ስላባቃ ወደ ቤት ለመሄድ ፈለገ/ች። በመሆኑም አዋላጅ ነርሱ/ሷ ታካሚዋን ወደ ሌላ ለመድረስ አንድ ሰአት የሚወስድ ጤና ተቋም እንድትሄድ ወሰነች።

ይህንን የአዋላጅ ነርስ እርምጃ እንዴት ትገመግመዋለህ/ሚዋለሽ?

1	2	3	4	5	6	7	8	9	10

በጣም እቃወማለሁ በጣም ደግፉለሁ

409. አንዷት ሴት ተንበርክካ መውለድ ፈለገች ነገርግን አዋላጅ ነርሱ/ሷ አልጋው ለዚህ አይሆንም ብሎ/ላ ያምናል/ታምናለች። ስለዚህ አዋላጅ ነርሱ/ሷ ተኝታ እንድትወልድ ያስገድዳታል/ታስገድዳታለች።

ይህንን የአዋላጅ ነርስ እርምጃ እንዴት ትገመግመዋለህ/ሚዋለሽ?

1	2	3	4	5	6	7	8	9	10

በጣም እቃወማለሁ በጣም ደግፉለሁ

410. አዋላጅ ነርሱ/ሷ ለወሊድ አገልግሎት ወደ ጤና ተቋም የመጣች ሴት በመርዳት ለይ ይገኛል/ትገኛለች። ሌሎች እናቶች በዚሁ ክፍል ውስጥ በወሊድ ለይ ናቸው። ሴቷ እያለቀሰችና እየጮሀች ሌሎቹን እየረበሸች ነው። የጤና ባለሙያዎቹ ስራቸውን በትኩረት መወጣት አልቻሉም። አዋላጅ ነርሱ/ሷ ድምጿን እንድትቀንስ ይነግራታል/ትነግራታለች ነገርግን ሴቷ መጮህ ትቀጥላለች። በመሆኑም አዋላጅ ነርሱ/ሷ በቁጣና በሀይለ ቃል ድምጿን እንድትቀንስ ይነግራታል/ትነግራታለች።

ይህንን የአዋላጅ ነርስ እርምጃ እንዴት ትገመግመዋለህ/ሚዋለሽ?

1	2	3	4	5	6	7	8	9	10

በጣም እቃወማለሁ በጣም ደግፉለሁ

ክፍል 5

በትምህርት ወቅት ስለተመለከቱት ወይም ስለተሳተፍበት የወሊድ አገልግሎት የተወሰነ ጥያቄ መጠየቅ እንፈልጋለን።

#	የጤና ባለሙያ የሚከተሉትን ሲያደርጉ ታስታውሳለህ/ሽያለሽ	1= በጭራሽ	2= አልፎአልፎ	3= አንዳንዴ	4= በብዛት
1	በምጥ ለይ ያለችን እናት እጅ ይይዛሉ?	1	2	3	4
2	በምጥ ለይ ያለችን እናት በጥፊ በመምታት እንድታምጥ ያበረታታሉ?	1	2	3	4
3	በምጥ ለይ ያለችን እናት ቤተሰቦች በወሊድ ወቅት እንዲሳተፉ ይጠይቃሉ?	1	2	3	4
4	ቤተሰቦች በወሊድ ወቅት እንዲወጡ ይጠይቃሉ?	1	2	3	4
5	በምጥ ለይ ያለች እናት ለይ በመጮህ እና በቁጣ እንድታምጥ ያበረታታሉ?	1	2	3	4
6	በምጥ ለይ ያለችን እናት መልካም እያደረገች እንደሆነ ይነግራሉ?	1	2	3	4
7	በምጥ ለይ ያለችን እናት ድምጿን እንድትቀንስ ይጠይቃሉ?	1	2	3	4
8	በምጥ ለይ ያለችን እናት ለረጅም ሰአት ለብቻዋ ይታዋታል?	1	2	3	4
9	በምጥ ለይ ያለችን እናት የሚያስፈልጋት ነገር ካለ ይጠይቋታል?	1	2	3	4
10	ክፍያ መፈጸም ያልቻለች እናትን ወይም ልጅዋን እንዳይወጡ ይከለክላሉ?	1	2	3	4
11	በምጥ ለይ ያለችን እናት አክብሮት በጎደለው ሁኔታ ይናገራሉ?	1	2	3	4
12	በምጥ ለይ ላለች እናት ምን ሊፈጠር እንደሆነ ያብራሉ?	1	2	3	4
13	በምጥ ለይ ያለችን እናት ጥያቄ ካላት ይጠይቋታል?	1	2	3	4
14	ሀብታም እናቶችን ከድሀ እናቶች የበለጠ ያክማሉ?	1	2	3	4
15	የተማሩ እናቶችን ካልተማሩ እናቶች የበለጠ ያክማሉ?	1	2	3	4
16	በምጥ ለይ ያለችን እናት ከመመርመር በፊት ፍቃደኝነቷን ይጠይቃሉ?	1	2	3	4
17	በምጥ ለይ ያለችን እናት ለወሊድ የሚያስፈልግ ነገሮችን ካላመጣች ይቆጥዋታል?	1	2	3	4
