# Supplementary material for: Development and use of a scale to assess gender differences in appraisal of mistreatment during childbirth among Ethiopian midwifery students
Source: PLoS One. 2020 Jan 16;15(1):e0227958. doi: 10.1371/journal.pone.0227958 (PMC6964878; doi:10.1371/journal.pone.0227958)
Supplement: S1 Table — (DOCX) [file pone.0227958.s005.docx]

|  | 1 | 2 | 3 | 4 | 5 | 6 | 7 | 8 | 9 | 10 |
| --- | --- | --- | --- | --- | --- | --- | --- | --- | --- | --- |
| 1 | 1.00 | .27 | .21 | .17 | .15 | .17 | .07 | .23 | .17 | .26 |
| 2 | .27 | 1.00 | .31 | .24 | .19 | .31 | .16 | .23 | .22 | .28 |
| 3 | .21 | .31 | 1.00 | .30 | .29 | .33 | .07 | .28 | .19 | .25 |
| 4 | .17 | .24 | .30 | 1.00 | .30 | .44 | .12 | .43 | .19 | .21 |
| 5 | .15 | .19 | .29 | .30 | 1.00 | .52 | .04 | .46 | .17 | .33 |
| 6 | .17 | .31 | .33 | .44 | .52 | 1.00 | .11 | .63 | .14 | .30 |
| 7 | .07 | .16 | .07 | .12 | .04 | .11 | 1.00 | .14 | .24 | .21 |
| 8 | .23 | .23 | .28 | .43 | .46 | .63 | .14 | 1.00 | .11 | .28 |
| 9 | .17 | .22 | .19 | .19 | .17 | .14 | .24 | .11 | 1.00 | .27 |
| 10 | .26 | .28 | .25 | .21 | .33 | .30 | .21 | .28 | .27 | 1.00 |
